# Treatment of Aseptic Necrosis of the Lunate Bone (Kienböck Disease) Using a Nickel–Titanium Memory Alloy Arthrodesis Concentrator: A Series of 24 Cases

**DOI:** 10.1097/MD.0000000000001760

**Published:** 2015-10-23

**Authors:** Yongqing Xu, Chuan Li, Tianhua Zhou, Yongyue Su, Xiaoqing He, Xinyu Fan, Yueliang Zhu

**Affiliations:** From the Department of Orthopedic Surgery, Kunming General Hospital, Kunming, Yunnan, China (YX, CL, TZ, YS, XH, XF, YZ).

## Abstract

Avascular necrosis of the lunate bone (Kienböck disease) is caused by loss of blood supply of the bone. This study aimed to evaluate the efficacy and safety of a novel nickel–titanium (Ni–Ti) memory alloy arthrodesis concentrator in the treatment of this disease.

A consecutive 24 patients with stage IIIb aseptic lunate necrosis were treated with scapho-trapezio-trapezoeid (STT) arthrodesis using a Ni–Ti arthrodesis concentrator from August 2008 to December 2012. Wrist pain, grip strength, carpal height, and scapholunate angle were measured and compared before and after the surgery. The wrist functions were evaluated using the Mayo scale.

Patients were followed up for a mean of 12 months (range, 6–24 months). Grip strength of the affected side was significantly improved after the surgery (18 ± 4.74 kg vs. 30.21 ± 7.14 kg, *P* < 0.0001). Wrist pain score was significantly decreased from 5.88 ± 0.9 to 0.5 ± 0.51 (*P* < 0.0001). Carpal height and Mayo score were also significantly increased after the surgery (*P* < 0.0001). Scapholunate angle was significantly decreased after the surgery (68.38 ± 7.28° vs. 49.91 ± 4.28°, *P* < 0.0001). No implant breakage, loose implant, wound infection, or nonunion occurred.

STT arthrodesis is effective for the treatment of stage IIIb lunate necrosis. The Ni–Ti memory alloy arthrodesis concentrator is a convenient tool for STT arthrodesis with excellent and reliable results.

## INTRODUCTION

Kienböck disease is a condition of the lunate bone caused by avascular, aseptic necrosis.^1,2^ The development of Kienböck disease is divided into 4 stages,^2^ which is used to guide treatment and to enable comparison of clinical outcomes (Table 1).

**TABLE 1:**
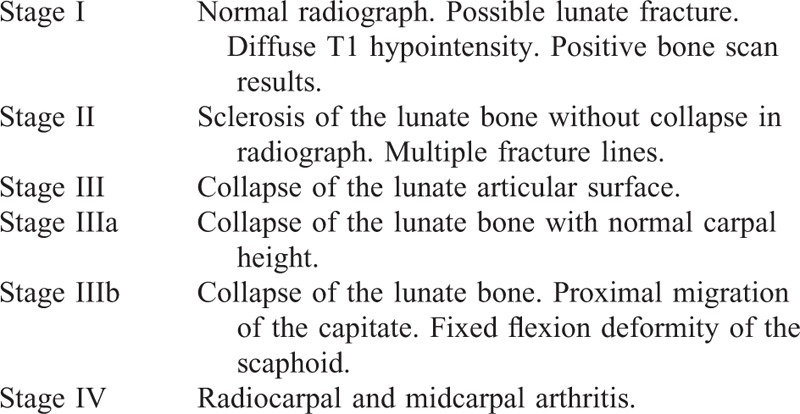
Stages of Kienböck Disease

Stage III is an advanced stage of Kienböck disease and its treatment is a clinical challenge. Various methods have been proposed for the treatment of Kienböck disease, such as transposition of pedicled pisiform bone and lunate bone replacement with pedicled scaphoid.^3^ An arthroscopic technique utilizing bone morphogenetic protein has also been used to reconstruct the necrotic lunate bone.^4^ Scaphocapitate arthrodesis^5,6^ and scapho-trapezio-trapezoeid (STT) arthrodesis^7,8^ have shown stable results in treating Kienböck disease in a long-term follow-up. Arthrodesis can decrease the load on the lunate bone and thus mitigate the necrosis by preventing the movement between the lunate bone and the distal row of the carpal bones.

**FIGURE 1:**
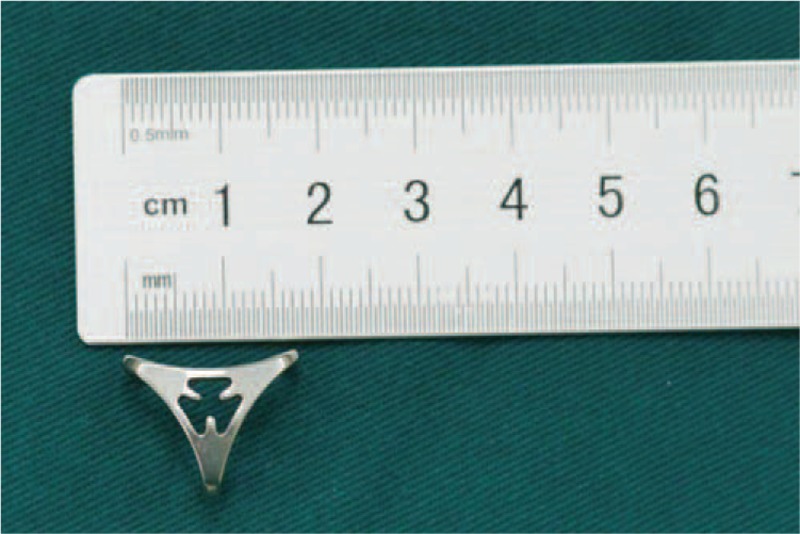
The Ni–Ti memory alloy scapho-trapezio-trapezoeid arthrodesis concentrator (top view).

We have designed a novel nickel–titanium (Ni–Ti) memory alloy STT arthrodesis concentrator for the treatment of Kienböck disease (Fig. 1). The concentrator consists of a triangle body and 3 arms forming an equilateral triangle. In the original shape of the concentrator, the arm–body angles are 70° inward. At 0 to 4°C, the arms are bent outwardly to increase the angle to 90°C. After inserting the arms into the scaphoid, trapezium, and trapezoid, the concentrator is warmed to 35 to 40°C. At the higher temperature, the concentrator returns to its original shape and the arm–body angle is decreased, resulting in constant inward pressure and holding the 3 bones tightly.

We have treated 24 patients with stage IIIb Kienböck disease using this Ni–Ti memory alloy STT arthrodesis concentrator and have achieved excellent results.

## METHODS

### Patients

From August 2008 to December 2012, a consecutive 24 patients with stage IIIb Kienböck disease were treated with STT arthrodesis using the Ni–Ti memory alloy concentrator at our hospital. The patients included 17 men and 7 women with a mean age of 38 years (range, 26–55 years). The right wrist was affected in 16 patients and left in 8 patients. This study was approved by the Institutional Review Board of Kunming General Hospital. Each of the 24 patients has signed a written informed consent form.

### Surgical Procedure

General anesthesia or brachial plexus block was used. The patient was put into the supine position and a tourniquet was used. A 3.5–4.0-cm dorsal transverse incision was carried out upon the scaphoid, trapezium, and trapezoid, distal to the styloid process. The annular ligament of carpus was incised along the extensor pollicis longus tendon. The wrist capsule was cut open between the extensor carpi radialis longus tendon and the extensor carpi radialis brevis tendon. The articular surfaces between the scaphoid, trapezoid, and trapezium were exposed. The distal part of the scaphoid was released. The lunate was adjusted to the neutral position and the scapholunate angle was maintained at 47°. The scaphoid, trapezium, and trapezoid were each temporarily held with a single K-wire. The cartilage and subchondral cortical bone of the scaphoid, trapezoid, and trapezium were removed. Allograft cancellous bone (Xinkangchen Medical Technology, Beijing, China) was packed into the spaces between the bones.

**FIGURE 2:**
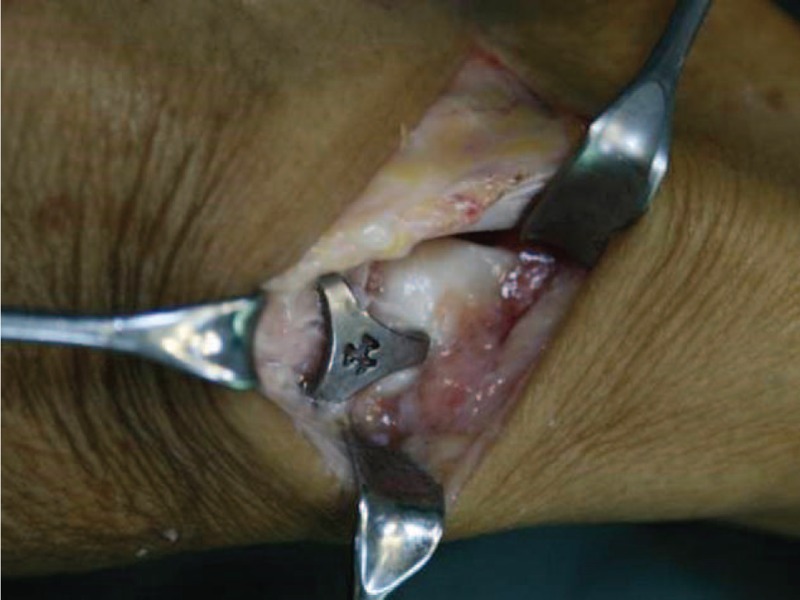
Intraoperative view of the Ni–Ti memory alloy concentrator after insertion into the scaphoid, trapezium, and trapezoid.

The concentrator was immersed in 0 to 4°C iced water for 10 minutes. The arm–body angle was increased to 90° by bending the arms outwardly. A 2-mm K-wire was used to drill 3 holes perpendicular to the bone surface of the scaphoid, trapezium, and trapezoid. Then, the concentrator was implanted by inserting the 3 arms into the holes in the bones. The holding K-wires were removed. While protecting the surrounding soft tissues with gauze, the body of the concentrator was heated using 35 to 40°C normal saline for 3 minutes. The concentrator was returned to its original shape, exerted inward pressure in the 3 carpal bones, and held tightly (Fig. 2). Fixation stability was check by rotating the wrist. The scapholunate angle and implant position were examined using C-arm fluoroscopy. The wound was closed in layers.

### Postoperative Management and Evaluation

**FIGURE 3:**
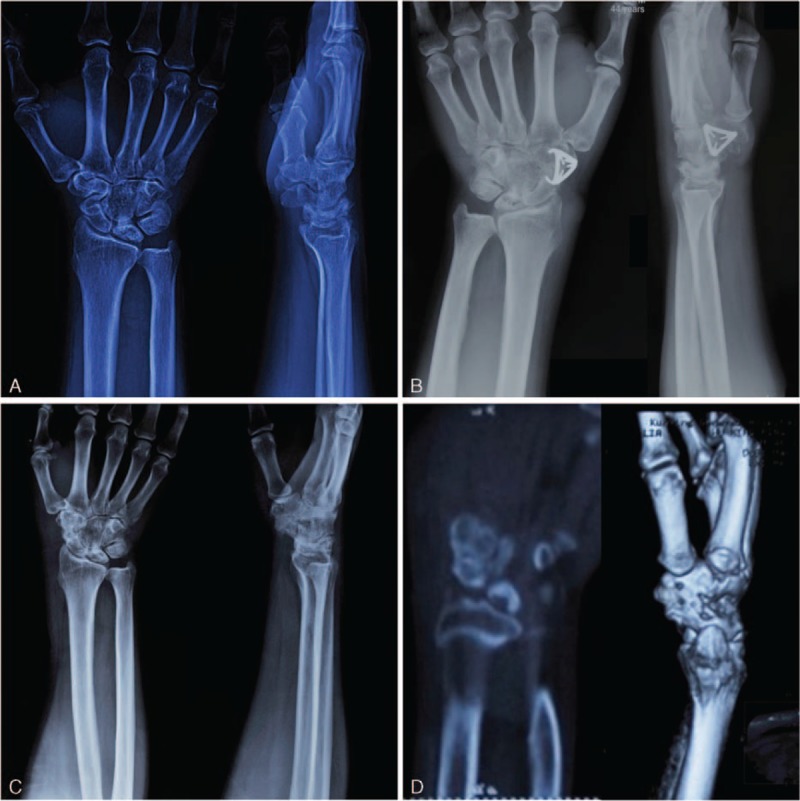
Radiographs of a 44-year-old man. A, Preoperative X-ray images showing rotation and laxation of the lunate bone, and increased scapholunate angle. B, Postoperative X-ray images showing the reduced lunate bone, normal scapholunate angle, and position of the implant. C, X-ray images after implant removal showing fusion of the scaphoid, trapezium, and trapezoid, and no further collapse of the lunate bone. D, Computed tomography images after implant removal showing the fused scaphoid, trapezium, and trapezoid.

Postoperative radiographs were taken to show the position of the implants. Bone fusion was confirmed using X-ray (Fig. 3A–C) and computed tomography (CT) (Fig. 3D) examination. X-ray examination was performed on postoperative day 7, and at 3 and 6 months. Thereafter, CT scan may also be used to evaluate the bone fusion condition. No external fixation was used in any patients. Wrist exercise was initiated from postoperative 6 weeks. The concentrator was removed at postoperative 6 months or later, up to 18 months in 1 patient. Carpal height and scapholunate angle were measured in radiographs. Grip strength was measured using a Jamar dynamometer (Sammons Preston Rolya, Bolingbrook, IL). Wrist pain was evaluated using the visual analogue scale. Wrist functions were assessed using the Mayo scale.^9^

### Statistical Analysis

All data were presented as mean ± standard deviation. Comparisons were made using paired Student *t* test. Statistical analyses were performed using SPSS 19.0 (SPSS Inc, Chicago, IL). *P *< 0.05 was considered statistically significant.

## RESULTS

**FIGURE 4:**
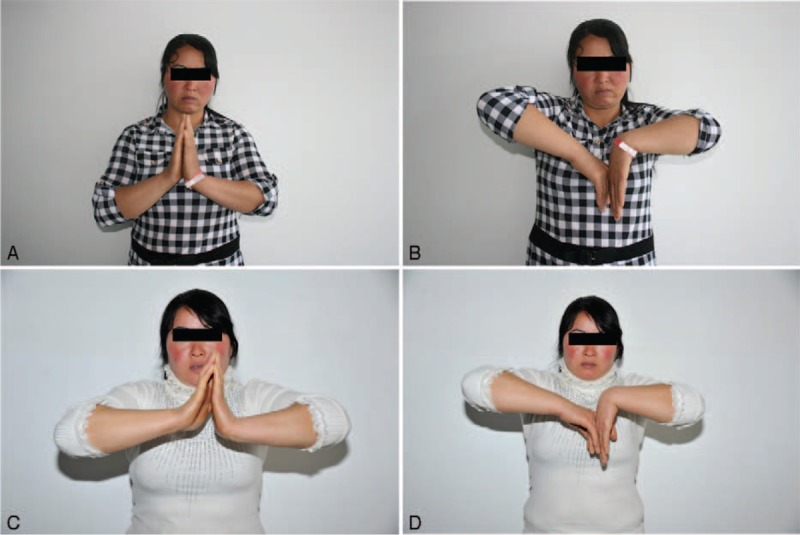
Range of wrist flexion and extension of a 34-year-old woman with her right hand affected. A and B, The preoperative ranges of wrist flexion and extension were severely restricted. C and D, After the surgery, the extension range was significantly improved, while the flexion range was mildly improved.

The STT arthrodesis significantly improves the wrist conditions in terms of lunate necrosis and scapholunate angle (Fig. 3). At postoperative 12 months, the scaphoid, trapezium, and trapezoid fused completely. Patients were follow up for a mean of 12 months (range, 6–24 months). No complications occurred such as loose implant, implant breakage, infection, and nonunion. Grip strength, wrist pain, carpal height, scapholunate angle, and Mayo scores of the affected side were all significantly improved after the surgery (Table 2). According to the Mayo score, 21 patients (87.5%) obtained excellent and good wrist functions after the surgery. Ranges of wrist flexion and extension were also significantly improved after the surgery (Fig. 4). The detailed patient information and evaluation results are listed in Supplementary Table 1, http://links.lww.com/MD/A471.

**TABLE 2:**
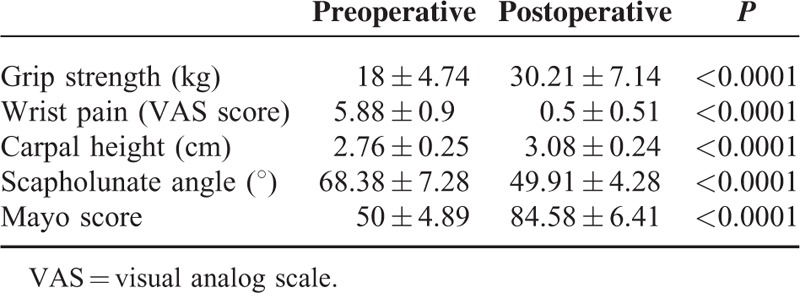
Evaluation of the Affected Wrists in 24 Patients (n = 24)

## DISCUSSION

Kienböck disease is caused by blood supply disruption and concentrated stress of the lunate bone.^10^ This disease is best treated with different strategies according to the disease stage.^11^ Kienböck disease of stage I is managed using plaster fixation. Stage II and IIa disease can be treated using various surgical procedures such as radius shortening, ulna extension, and capitate shortening. Stage IIIb disease can be treated using carpal bone fusion,^12,13^ lunate bone resection, radius shortening, and proximal carpal row resection.^14^ Stage IV disease is usually treated using proximal carpal row resection, total wrist fusion, and wrist denervation.^2^ However, there is still controversy about the best treatment methods of Kienböck disease due to the lack of controlled studies.^14,15^

After STT arthrodesis, load is conducted through the fused scaphoid, trapezium, and trapezoid onto the normal joints, thus re-establishing the wrist stability. STT arthrodesis does not affect the articulation between the triquetral bone and the hamate bone, and has no effects on the roles of this joint in wrist movement, thus preserving partial movement range of the wrist. STT arthrodesis also transfers part of the radial lunate joint load to the radio-scaphoid joint, and significantly reduces the lunate bone load, thus promoting the regeneration of the necrotic bone.^16^

Various implants have been used in the partial carpal bone fusion surgeries with their own pros and cons. K-wires are inexpensive and easy for manipulation, but do not produce compressive forces and are associated with trajectory infection. Herbert screws damage the surrounding normal articular surface, and have a limited compressive force. In addition, Herbert screws are expensive, need real-time intraoperative fluoroscopy, and have a long learning curve. Although U-shaped nails can be easily manipulated, they produce limited compressive force. U-shaped nails require 2 to 3 cross fixations and additional postoperative plaster immobilization of the wrist, which can hinder the postoperative functional recovery.

In comparison to the previously used implants, our Ni–Ti memory alloy arthrodesis concentrator is easy to use, and can produce adequate compressive forces. The body of the concentrator is designed with a hollow center, through which the fusion results can be observed in the postoperative X-ray images. However, there is a concern of cortex breach if the concentrator arms are inserted too closely to the cortex. By using concentrators of proper size and centering the holes in each carpal bone, we have not observed cortex breach caused by the concentrator arm in these 24 patients. C-arm fluoroscopy examination is needed after insertion of the concentrator to find out any signs of cortex breach.

Our study has limitations. First, the follow-up time is relatively short, which may be inadequate to see all the complications to unfold. Second, only one evaluation time point was available.

In conclusion, this novel Ni–Ti memory alloy STT arthrodesis concentrator is safe and effective for the treatment of stage IIIb Kienböck disease. Excellent surgical outcomes were achieved using this arthrodesis concentrator without significant complications.
